# Recurrent Inflammatory Panniculitis with Partial Lipoatrophy and Elevated temperature: a possible new autoinflammatory disorder

**DOI:** 10.1186/1546-0096-13-S1-P171

**Published:** 2015-09-28

**Authors:** A Torrelo, L Noguera-Morel, A Hernández-Martín, D Clemente, H Kutzner, JM Barja, A Almeida de Jesus, JC López-Robledillo, R Goldbach-Mansky, L Requena

**Affiliations:** 1 Hospital Niño Jesús, Dermatology, Madrid, Spain; 2 Hospital Niño Jesús, Rheumatology, Madrid, Spain; 3 Dermatohistopathologisches Gemeinschaftslabor, Pathology, Friedrichshafen, Germany; 4 Hospital del Bierzo, Dermatology, Ponferrada, Spain; 5 NIH, Translational Autoinflammatory Disease Section, Bethesda, USA; 6 Fundación Jiménez Díaz, Dermatology, Madrid, Spain

## Question

We report a series of cases with recurrent episodes of inflammatory subcutaneous nodules followed by fat atrophy in the affected area in children with fever, malaise, abdominal pain, hepatosplenomegaly, and some laboratory abnormalities that often persist beyond the febrile attacks of panniculitis.

## Patients

Five children with the above mentioned clinical features are presented. Skin histopathology with a panel of immunohistochemistry markers was obtained from all patients.

## Results

Histopathology showed a mostly lobular panniculitis, without vasculitis, with a mixed inflammatory infiltrate with evolving prominent cellularity with neutrophils, then lymphocytes and finally histiocytes. Lipophagia was considered the cause of lipoatrophy. On immunohistochemistry, T-lymphocytes, both CD4 and CD8, were strongly represented, with a higher proportion of CD8. Myeloperoxidase (MPO) staining was positive in the infiltrate in all cases. MPO-positive cells were mainly located surrounding individual adipocytes. CD68/PGM1 stain was positive in all cases, and the infiltrate was more prominent in late lesions, showing prominent lipophagia. CD68/PGM1 cells were located within the infiltrate, but were also strikingly distributed around individual adipocytes. Double staining with MPO and CD68/PGM1 showed that MPO-positive and CD68/PGM1 cells were different, thus indicating that in early stages MPO-positive cells induce damage to adipocytes and later, CD68/PGM1 macrophages will phagocyte adipocytes, leading to lipophagia and lipoatrophy. We found intensely positive stain for STAT1 in all cases, whereas STAT2 was negative in all samples. Only the cells in the inflammatory infiltrate in the fatty lobules were stained for STAT1.

## Conclusion

We speculate that this allegedly new disease is possibly one autoinflammatory disease with enhanced IFN-I signaling.

